# Response of tomato wilt pathogen *Ralstonia solanacearum* to the volatile organic compounds produced by a biocontrol strain *Bacillus amyloliquefaciens* SQR-9

**DOI:** 10.1038/srep24856

**Published:** 2016-04-22

**Authors:** Waseem Raza, Ning Ling, Liudong Yang, Qiwei Huang, Qirong Shen

**Affiliations:** 1 Jiangsu Collaborative Innovation Center for Solid Organic Waste Utilization, College of Resources and Environmental Sciences, Nanjing Agricultural University, Wei Gang Road, No. 1, 210095, Nanjing, Jiangsu Province, P.R. China

## Abstract

It is important to study the response of plant pathogens to the antibiosis traits of biocontrol microbes to design the efficient biocontrol strategies. In this study, we evaluated the role of volatile organic compounds (VOCs) produced by a biocontrol strain *Bacillus amyloliquefaciens* SQR-9 on the growth and virulence traits of tomato wilt pathogen *Ralstonia solanacearum* (RS). The VOCs of SQR-9 significantly inhibited the growth of RS on agar medium and in soil. In addition, the VOCs significantly inhibited the motility traits, production of antioxidant enzymes and exopolysaccharides, biofilm formation and tomato root colonization by RS. The strain SQR-9 produced 22 VOCs, but only nine VOCs showed 1–11% antibacterial activity against RS in their corresponding amounts; however, the consortium of all VOCs showed 70% growth inhibition of RS. The proteomics analysis showed that the VOCs of SQR-9 downregulated RS proteins related to the antioxidant activity, virulence, carbohydrate and amino acid metabolism, protein folding and translation, while the proteins involved in the ABC transporter system, amino acid synthesis, detoxification of aldehydes and ketones, methylation, protein translation and folding, and energy transfer were upregulated. This study describes the significance and effectiveness of VOCs produced by a biocontrol strain against tomato wilt pathogen.

The plant growth promoting rhizobacteria (PGPR) living in the rhizosphere exert beneficial effects on the plants by interacting with roots as well as with other microbes in the rhizosphere^1^. On the other hand, deleterious microbes living in the rhizosphere cause plant diseases which sometimes results in the complete loss of crops^2^. Some of the PGPRs have ability to prevent the development of plant soil-borne diseases by keeping the level of deleterious microbes below the threshold limit. These PGPRs which are also called as biocontrol agents produce antibiotics, antimicrobial volatile organic compounds (VOCs) and hydrolytic enzymes and improve the resistance of plants against different pathogens. These biocontrol agents are isolated and introduced in an optimum quantity in the rhizosphere to control the development of plant diseases. The biocontrol of soil-borne diseases of plants has appeared as a striking environment friendly substitute to other control methods like crop rotation, use of chemicals, soil management practices and resistant plant cultivars^3^.

Among different plant diseases, bacterial wilt caused by *Ralstonia solanacearum* (RS) is a devastating disease of crops. This disease occurs widely in tropical and subtropical regions of the world and causes severe yield losses of many important crops like tomato, eggplant, potato, tobacco and pepper as well as other important crops like banana, peanut and ginger^4^. There are almost 200 different plant species within 53 taxonomic families which have been reported the host of RS^5^. The pathogen colonizes the root surface and then invades xylem vessel by degrading cell wall and produces large amounts of exopolysaccharides that block water flow causing chlorosis, wilting and ultimately plant death. In addition, RS is difficult to eliminate completely because it can persist in the soil for long periods along with infested plant debris^6^. Different approaches, for example, resistant plant varieties, cultural practices, crop rotation and chemicals, etc. have been used to overcome this disease, but all methods showed limited success. The diverse genetic diversity of RS overcomes the resistance of the crops so it is highly recommended to identify the biovar and race of the pathogen before deciding control methods^7^.

Several bacterial biocontrol agents have been reported to control the RS of tomato including *Bacillus* spp., *Pseudomonas* spp., *Streptomyces* spp., *Paenibacillus* spp., and *Trichoderma* spp.^8^,^9^,^10^. Among those, *Bacillus* species, particularly *B. subtilis*, *B. cereus*, and *B. amyloliquefaciens* are most effective in controlling plant diseases^10^. In addition, *Bacillus* species are considered excellent for the formulation of commercial products because of their ability to form spores that can survive in a wide range of environments^11^. The selection of biocontrol agents is a systematic work and requires preliminary experiments *in vitro* and *in vivo* to evaluate their true potential to control the RS. Normally, a biocontrol agent should have the ability to colonize plant roots efficiently with diverse modes of action that can hit pathogen with multiple weapons like the production of a diverse array of antibiotics, hydrolytic enzymes and VOCs^11^,^12^. Among the different modes of action of biocontrol agents, the production of VOCs has its own importance. The VOCs not only can control pathogen in the rhizosphere but also have ability of long distance control due to their volatile nature. The VOCs have not only been reported to display antifungal, antibacterial and nematicidal activity, but also have been reported as plant growth promoting and systemic resistance inducing agents in plants^2^,^3^. The VOCs produced by *B. amyloliquefaciens* PPCB004 especially 3-hydroxy-2-butanone inhibited the mycelial growth of *Penicillium digitatum* Sacc., *P. italicum* Wehmer and *P. crustosum* Thom and this effect was up to 73.3% against *P. crustosum*^13^. Similarly, the VOCs produced by *B. subtilis* GB03 and *B. amyloliquefaciens* IN937a promoted the growth of *Arabidopsis thaliana*^14^. *Pseudomonas fluorescens* B-4117 and *Serratia plymuthica* IC1270 produced VOCs that inhibited the growth of *Agrobacterium tumefaciens* and *Agrobacterium vitis*^15^.

However, there is no information about the effect of VOCs produced by the biocontrol strains on the growth and pathogenicity traits of RS. The objective of this study was to evaluate the effects of VOCs produced by a biocontrol strain *B. amyloliquefaciens* SQR-9 on the growth and pathogenicity traits of tomato bacterial wilt pathogen RS on agar medium and in soil using biochemical and proteomics techniques. The strain SQR-9 has shown antagonist effects against a number of plant pathogens^8^,^15^. The pathogenicity traits of RS evaluated in this study were motility traits, hydrolytic enzymes, antioxidant activity, exopolysaccharides, biofilm formation and root colonization.

## Methods

### Microbial strains

The biocontrol strain *B. amyloliquefaciens* SQR-9 (China General Microbiology Culture Collection Center, CGMCC accession no. 5808), isolated from the cucumber rhizosphere, was provided by our laboratory^8^. The bacterial pathogenic strain *R. solanacearum* ZJ3721 (biovar 3) (RS) which exhibited high virulence in tomato was kindly provided by Dr. Jian-Hua Guo (Department of Plant Pathology, Nanjing Agricultural University). The nutrient agar (NA) medium was used to grow strain SQR-9 while the RS strain was grown on tetrazolium chloride agar medium as a working stock and on casamino acid-peptone-glucose (CPG) agar medium for experiments^15^. Both strains were stored in their respective medium supplemented with 30% glycerol at −80 °C.

### Antibacterial activity assay of VOCs produced by SQR-9 on agar media

The effect of VOCs produced by strain SQR-9 on the growth of RS was evaluated in divided petri plates (85 mm diameter). The overnight cultures of strain SQR-9 in NA medium and of strain RS in CPG medium were washed twice and resuspended in sterilized water up to the concentration of 1 × 10^7^ colony forming units (CFU)/ml (~equal to OD_600_ 0.1 for SQR-9 and OD_600_ 0.01 for RS). Two 5 μl cell suspensions of strain SQR-9 were spotted onto the one compartment of divided plates containing modified minimal salt (MS) medium (1.5% agar, 1.5% sucrose, and 0.4% TSA (w/v)) and two 5 μl cell suspensions of strain RS were spot inoculated on to the other compartment of the divided plates containing CPG agar medium. The plates were sealed with parafilm and incubated at 30 °C. Four control treatments included: First control without the inoculation of strain SQR-9 (the plates were not sealed to access the normal growth of RS), the second control without the inoculation of strain SQR-9 (the plates were sealed to expose RS to its own VOCs), the third control containing *Escherichia coli* DH5α in place of strain SQR-9 to determine whether the decrease in the growth of RS was because of oxygen depletion consumed by two microbes in sealed plate or not, and in the fourth control, activated charcoal (2 g) was placed between two compartments of divided plates spot inoculated with strain SQR-9 and RS, respectively. For the placement of activated charcoal, 0.5 cm agar medium from the edge of both sides of the plate dividing point was carefully removed and the empty place was filled with the activated charcoal. After each 12 hours up to five days, the colonies of strain RS along with agar medium were removed from the divided plates, suspended in 1 ml sterilized water, diluted by 1:500 (2 × 10^−3^) and spread on the CPG agar medium. The CFU/ml of RS was determined after two days incubation at 30 °C. To check the bacteriostatic or bactericidal role of VOCs of SQR-9, three plates were placed back at 30 °C after removing parafilm and strain SQR-9 along with agar medium from one side of the divided plates.

The effect of VOCs produced by different concentrations of strain SQR-9 on the RS was determined by spot inoculation (5 μl) of water suspended cells of strain SQR-9 at 1, 5 and 10 places and spread (100 μl) onto the one compartment of the divided plates except control plates. While the other compartment was spot inoculated with water suspended cells of RS (1 × 5 μl) and after three days, RS colony counts on the CPG agar medium (CFU/ml) were determined as described earlier. Similarly, the effect of VOCs produced by strain SQR-9 was evaluated on the different initial concentrations of RS by diluting its water suspended cells (10^7^ CFU/ml) by 0, 2, 5, 10 and 20 times and spotted onto one compartment of divided pates (2 × 5 μl) while the other compartment was spot inoculated (2 × 5 μl) with strain SQR-9 as described earlier except control plates. The colony counts of RS on the CPG agar medium (CFU/ml) were determined as described earlier.

The antibacterial potential of VOCs of SQR-9 grown on modified MS medium was evaluated on RS present in the diseased soil. For that, SQR-9 was inoculated onto the modified MS medium as described earlier, except control plates and other compartment was added with 7.5 g (dry weight) of tomato diseased field soil (5 × 10^5 ^CFU/ml) collected from Yixing, China. The plates were sealed with parafilm and after 14 days incubation at 30 °C, RS colony counts in infested soil were determined using the dilution plate technique on CPG agar medium supplemented per liter with 50 mg 2, 3, 5-tripheny tetrazolium chloride, 5 mg crystal violet, 100 mg polymyxin B sulfate, 25 mg bacitracin, 5 mg chloromycetin, 0.5 mg penicillin and 100 mg cycloheximide^16^.

The ammonia and cyanide production assays were conducted because production of those by strain SQR-9 could be a reason of antibacterial activity against RS^17^,^18^. For that, strains SQR-9 and RS were spot inoculated (2 × 5 μl) onto the modified MS medium in divided petri plates as described earlier. For the ammonia production assay, the modified MS medium for strain RS was added with 0.02 g/L of bromthymol blue dye and for the cyanide production assay, a picrate/Na_2_CO_3_ paper strip was fixed to the underside of the divided petri plate lid. In control, strain SQR-9 was not inoculated onto the one compartment of divided plates. Later, the plates were sealed with parafilm and incubated at 30 °C. After three days, for the ammonia production assay, the change in the color of medium supplemented with bromthymol blue dye was observed while for the cyanide production assay, a color change of paper strip from yellow to brown, or reddish brown was recorded as compared to control, respectively^17^. The colony counts of RS on the CPG agar medium (CFU/ml) were also determined to evaluate its growth inhibition as described earlier. All the experiments described here had three replicates and were repeated twice.

### Antibacterial activity assay of VOCs produced by SQR-9 in soil

The antibacterial activity assay of VOCs produced by strain SQR-9 in natural soil and sterilized soil was conducted in divided plates. The healthy soil (pH 6.5, organic matter content 11.65 g/kg, and available N, P, K contents 41.3, 238.7, 177.5 mg/kg, respectively) was taken from a field in Yixing, China. The overnight culture of strain SQR-9 was suspended in sterilized water, diluted to 10^7 ^CFU/ml and 1 ml was mixed with 7.5 g (dry weight) natural or sterilized soil (121 °C for 30 min) and added into the one compartment of divided plates, while the other compartment was spot inoculated with strain RS (2 × 5 μl) onto the CPG agar medium as described earlier. In control, natural soil or sterilized soil was added into the one compartment of divided plates without the inoculation of strain SQR-9. The plates were sealed with parafilm and incubated at 30 °C. After three days, the CFU/ml calculation of RS was done on the CPG agar medium as described earlier. This experiment had five replicates and was repeated twice.

### Motility traits assay

The effect of VOCs of strain SQR-9 was determined on the motility traits of RS. For that, the strain SQR-9 was spot inoculated onto one compartment of divided plates containing modified MS agar medium as described earlier except control. While for the other compartment, the overnight culture of RS was washed twice and resuspended in sterilized water (OD_600_ = 1.0), and 2 μl was spot inoculated onto the CPG medium containing 0.3%, 0.7% and 1.6% (w/v) agar for the swimming, swarming and twitching motilities, respectively. For swimming and swarming motility, the zones of migration were measured in four directions after three days incubation at 30 °C and expressed as an average diameter^19^. For twitching motility assay, the morphology of the colony edges was observed under a microscope (4 × magnifications) after two days incubation at 30 °C^19^.

For the chemotaxis assay, the other compartment was added with the chemotaxis buffer agar medium (10 mM phosphate buffer, 0.1 mM EDTA, 1 μM methionine, 10 mM lactic acid, 0.35% agar and pH 7.3). Later, a 5 mm of the agar plug was removed from the medium and the holes were filled with 100 μl of tomato root exudates filtered by Millipore membrane filter (0.22 μm) except negative control, where the holes were filled with 100 μl of sterilized water. The strain RS was spot inoculated (2 μl) at 15 mm edge to edge distance from the holes. The plates were sealed with parafilm and incubated at 30 °C^20^. The RS cells, which had moved towards root exudates, were removed after three days and the CFU/ml calculation of RS was done on the CPG agar medium as described earlier. The root exudates of tomato containing 8.1% carbohydrates and 1.5% protein were extracted after 30 days of plant growth as described by Raza *et al.*^21^. These experiments had three replicates and were repeated twice.

### Hydrolytic enzyme production assay

The effect of VOCs of SQR-9 on the hydrolytic enzyme production by RS was determined in divided petri plates. For that, one side of the plates was spot inoculated with strain SQR-9 on modified MS agar medium as described earlier, except control, while the other side was spot inoculated with 2 μl of water washed 10^10^ cells/ml of overnight culture of RS on MS medium (0.8% agar) supplemented with 1% sodium carboxymethyl cellulose for β-1,4-endoglucanase activity and with 0.125% polygalacturonic acid for polygalacturonase activity. After two days, β-1,4-endoglucanase activity was detected by staining with a Congo red solution (1 mg/ml) for 15 min followed by NaCl (1 M) solution for 15 min and polygalacturonase activity was determined by staining with 0.1% ruthenium red. The clearing zones around the colonies of RS were measured in four directions. This experiment had three replicates and was repeated twice.

### Antioxidant enzyme production assay

The production of antioxidant enzymes, catalase and superoxide dismutase (SOD), by pathogen RS after exposure to the VOCs of strain SQR-9, was determined using divided plates, which were prepared as same as for growth inhibition assay. After three days, the RS colonies were washed twice and suspended in sterilized water. Total cellular proteins were extracted by sonication with the output power of 30% and on/off bursts of 10 s up to 5 min. After sonication, the mixture was centrifuged for 30 minutes (12000 × g at 4 °C) and the supernatant was used to evaluate the antioxidant enzymes. Total protein was calculated by the Bradford method using bovine serum albumin as standard^22^. The catalase activity was determined using KMnO_4_ method as described by Chance & Maehly^23^ and defined as the amount of H_2_O_2_ (μM) broke down per min by one mg of protein. The SOD activity was determined using nitroblue tetrazolium (NBT) method and the amount of SOD enzyme that inhibited 50% of NBT was defined as one enzyme unit (U)^23^. This experiment had three replicates and was repeated twice.

### Tomato seedling root colonization, EPS production and biofilm formation assays

Surface sterilized tomato seeds (*Lycopersicon esculentum*, cv. Jiangshu) were germinated on wet filter paper in Petri plates at 30 °C and then grown in trays containing sterilized vermiculite. The seedlings were irrigated with half-strength Hoagland nutrient solution and after ten days, were used for further assay. The RS cell suspensions (10^7 ^CFU/ml) with and without exposure to the VOCs of SQR-9 were prepared as described earlier. The roots of ten days old tomato seedlings were immersed in 5 ml cell suspensions of RS at room temperature and after 30 min rinsed with sterile water and blotted slightly. Later, the roots were cut, weighed and ground in sterile water and serial dilution plated on CPG agar medium to quantify the total bacteria adhering to the roots. The cell numbers were normalized to the seedling’s root fresh weight.

The biofilm formation assay was conducted in divided petri plates. One side of the plates was used for the inoculation of the strain SQR-9 on MS agar medium while the other side was used to inoculate RS in CPG broth. For the inoculation of RS, water agar (1.5% agar) was poured onto the other side and 2 ml Eppendorf tubes cut up to 250 μl capacity was inserted in the hot water agar. Later, 190 μl CPG medium was added into it and inoculated with 10 μl of 10^7 ^CFU/ml of water washed RS overnight culture. The plates were sealed with parafilm and placed at 30 °C for 48 hours. Crystal violet staining and biofilm estimation was performed as described by O’Toole & Kolter^24^.

For the EPS estimation, the RS colonies with and without exposure to the VOCs of SQR-9 were suspended in sterilized water and after measuring OD_600_ and cell counts on the CPG agar medium as described earlier, were shaken at 40 °C for half an hour to solubilize EPS present in the solution and then centrifuged at 12,000 × g for 10 min. Later, two volumes of ice cold ethanol were added in the cell free culture and placed at 4 °C for 24 hours. The EPS pellets were obtained by centrifugation at 12,000 × g for 10 min, dissolved in sterilized water and dialyzed using a membrane of 1000 Da molecular weight cutoff against ultra-pure water at 4 °C to remove the small molecules and entrained media residues. After two days, the EPS content was quantified by the phenol-sulfuric acid method^25^. These experiments had three replicates and were repeated twice.

### Collection and identification of VOCs produced by strain SQR-9

The VOCs produced by SQR-9 were collected using solid-phase microextraction (SPME) fiber [Supelco (Bellefonte, PA) stable flex divnylbenzene/carboxen/polydimethylsiloxane (DCP, 50/30 μm)] in triplicate. For that, strain SQR-9 was inoculated onto the modified MS agar medium in a 100 ml vial except control and incubated at 30 °C. After three days, the SPME fiber was inserted into the vial and incubated at 50 °C. After 30 min, the SPME fiber was inserted in the injector of GC-MS (Trace DSQ, Finnigan) and desorbed at 220 °C (1 min) with an RTX-5MS column (30 m, 0.25-mm inside diameter, 0.25 μm). The protocol used for over temperature was 33 °C (3 min), 180 °C (10 °C/min) and 240 °C (35 °C/min), and held for 5 min. The mass spectrometer was operated in the positive electron ionization mode at 70 eV and 220 °C with a scan from 50 to 500 m/z. The mass spectra of VOCs were compared with those in the NIST/EPA/NIH Mass Spectrometry Library with respect to the spectra in the Mainlib and/or Replib databases. The production of identified VOCs by strain SQR-9 was further confirmed and quantified by comparing with standard compounds (0.2–20 μg) except furan 2-ethyl-5-methyl. The standards were also run as same as samples using SPME fibers. For that, appropriate amount of each standard was added in 100 ml vial and then the SPME fiber was inserted in it and incubated at 50 °C for 30 min. Later, the SPME fiber was inserted in the injector of GC-MS. The pure standard compounds were purchased from Sigma, Tokyo Chemical Industry Co., Ltd. (TCI, Japan) and Aladdin Reagent Database, Inc. (Shanghai, China).

To check the antagonistic effect of VOCs produced by strain SQR-9, the sterile filter paper discs containing detected amount of each VOC or consortium of all VOCs except control (methanol was used as a control) were placed onto one compartment of divided plate in triplicate while the other compartment was spot inoculated with 2 × 5 μl cell suspension of the RS on CPG agar medium. After three days incubation at 30 °C, the colony counts of strain RS (CFU/ml) were determined on the CPG agar medium as described earlier. The results were expressed as percent inhibition compared to control.

### Protein extraction and 2D electrophoresis

Total proteins of RS with and without exposure to the VOCs of strain SQR-9 were extracted after three days incubation at 30 °C as described earlier. FOCUS™ Bacterial Proteome (G-Biosciences) kit was used for the extraction of total protein according to the manufacturer’s instructions. The quantification of proteins was done by the Bradford method^19^. For 2D analysis, almost 20 μg proteins were diluted to 250 μl of rehydration buffer (7 M urea, 2 M thiourea, 2% CHAPS, 50 mM dithiothreitol and 0.5% (v/v) carrier ampholytes, pH 3–10 nonlinear) and was applied to 13 cm immobilized pH gradient (IPG) strip, pH 3–10 NL in IPG box. After 12 hours, IPG strips were placed in Ettan IPGphor 3 manifold and isoelectric focusing (IEF) was done with consecutive steps at 100 V for 1 h, 200 V for 1 h, 500 V for 1 h, 1,000 V for 1 h and 8,000 V for 7.5 h. Later, IPG strips were kept at −80 °C or equilibrated immediately for 15 min in a buffer (50 mM Tris-HCl, pH 8.8, 6 M urea, 30% (v/v) glycerol, 2% (w/v) SDS) containing 2% (w/v) dithiothreitol and later in buffer containing 2.5% (w/v) iodoacetamide. For the second-dimension analysis, Dodeca Cell (Bio-Rad) with 12% acrylamide SDS-PAGE gels was used. Equilibrated IPG strips were put on top of 12% acrylamide SDS-PAGE gels, sealed with 0.5% agarose containing a trace amount of bromophenol blue, and second-dimension separation was performed by electrophoresis at 100 mA until the front had reached the lower end of the gel. Proteins were detected by silver staining and analyzed by ImageMaster 2D Platinum 7.0 software (GE Healthcare). The protein extraction and 2D analysis was conducted in triplicate.

### Mass spectrometry and Protein identification

The protein spots that showed more than two time expression difference were excised from the gel and destained for 10 min using 30 mM potassium ferricyanide and 100 mM sodium thiosulfate. Later, repeatedly washed with water and acetonitrile and digested in-gel with modified trypsin (Roche Diagnostics) in 50 mM ammonium bicarbonate. After digestion, peptides were concentrated using C18-ZipTips (Millipore) and eluted directly on the MALDI target in 1 μl of a saturated solution of α-cyanohydroxycinnapinic acid in 50% acetonitrile (vol/vol). Peptides were analyzed using a Voyager STR-DE MALDI-TOF mass spectrometer (Applied Biosystems) operated in reflectron mode at 20 kV accelerating voltage. The MALDI-TOF mass spectra were calibrated internally with known trypsin peaks, and proteins were identified by searching masses of measured peptides against *Ralstonia solanacearum* proteins in non-redundant protein databases using the peptide mass fingerprint tool in Mascot (Matrix Science) allowing a mass tolerance of 40 ppm.

### Statistical analysis

Student’s t-test (*P* = 0.05) was used to evaluate the significance between two treatments. One-way analysis of variance (ANOVA) was used for the evaluation of significance of more than two treatments and Duncan’s multiple-range test was employed to assess differences among treatments at *P* = 0.05 using SPSS ver. 19.0 statistical software (SPSS, Chicago, IL).

## Results

### Antibacterial activity of VOCs produced by SQR-9 on agar medium and in soil

**Figure 1:**
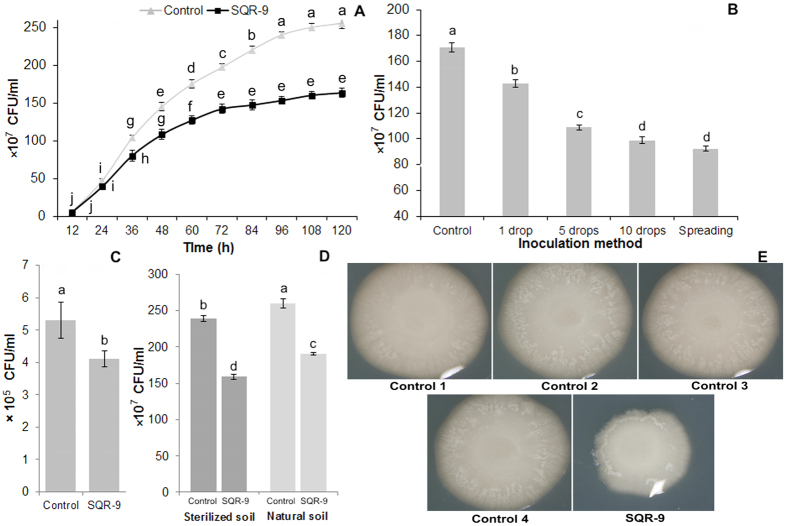
The antibacterial activity of volatile organic compounds (VOCs) produced by *Bacillus amyloliquefaciens* SQR-9 on agar medium and in soil against *Ralstonia solanacearum*. **(A)** Antibacterial activity of VOCs up to five days on agar medium, **(B)** antibacterial activity of VOCs produced by different inoculum concentrations of SQR-9, **(C)** antibacterial activity of VOCs produced by SQR-9 on agar medium against *Ralstonia solanacearum* in infested soil, **(D)** antibacterial activity of VOCs produced by SQR-9 in natural soil and sterilized soil against *Ralstonia solanacearum* inoculated on agar medium, **(E)** antibacterial activity of VOCs of SQR-9 on agar medium in divided plate assay. Control 1 = without the inoculation of strain SQR-9 (plates were not sealed); Control 2 = without the inoculation of strain SQR-9; Control 3 = *Escherichia coli* DH5α in place of strain SQR-9; Control 4 = activated charcoal was placed between two compartments of divided plates. All control treatments were inoculated with *Ralstonia solanacearum* and sealed with parafilm except control 1 and incubated at 30 °C for three days. Error bars indicate standard deviations of three replicates and different letters describe significant differences at *P* = 0.05 within the same data group.

The results showed that the VOCs produced by strain SQR-9 decreased the growth of RS, which reached 28% after three days and then reached 40% after five days (*P* = 0.0001) compared to second control (Fig. 1A). The third control treatment containing *E. coli* DH5α in place of strain SQR-9 showed similar results compared to second control (data not shown). In the fourth control, the use of activated charcoal to adsorb VOCs produced by strain SQR-9 eliminated their antibacterial effect on pathogen RS (data not shown). In the first control, where plates were not sealed to assess the difference of growth of RS with and without exposure to its own VOCs showed non-significant differences with second control. The production of ammonia and cyanide by SQR-9 was also tested and results showed that the color of agar medium containing bromthymol blue dye was not changed compared to control which indicated that strain SQR-9 was not able to produce ammonia (data not shown). Similarly, the yellow paper strip color was remained as same as control after three days incubation, which revealed that strain SQR-9 was also not able to produce cyanide. The plates, which were placed again at 30 °C after removing parafilm and strain SQR-9 along with agar medium from one side of divided plates, resumed normal growth rate. These results revealed that the VOCs of SQR-9 have bacteriostatic effect on the RS and the growth inhibition of RS was not because of oxygen depletion or ammonia/cyanide production, but was only because of the VOCs produced by strain SQR-9.

The VOCs produced by the increased inoculum concentrations of strain SQR-9 also increased the growth inhibition of pathogen RS. This increase in the growth inhibition of RS was 17%, 36%, 43% and 46% with 1, 5 and 10 drops inoculation and spreading inoculation methods (*P* = 0.0001) of strain SQR-9, respectively (Fig. 1B); however, it was nonsignificant between 10 spots inoculation and spreading inoculation methods. The reason might be that the strain SQR-9 showed spreading growth behavior on plates and after 10 drops inoculation onto the one compartment, it covered almost the whole surface of the agar medium after two days, which was similar to that of the spreading inoculation method. The results of the effect to VOCs of SQR-9 on the different initial inoculum concentrations of RS showed that the growth inhibition effect of VOCs was almost similar to the different inoculum concentrations of RS compared to the corresponding RS inoculum concentration control (data not shown). The antibacterial effect of VOCs of SQR-9 grown on the synthetic medium was evaluated on the RS present in the infected soil. The results showed that the VOCs produced by strain SQR-9 on synthetic medium decreased the population of RS by 23% (*P* = 0.013) in the infested soil after 14 days (Fig. 1C). The results about the antibacterial effect of VOCs produced by strain SQR-9 inoculated in sterilized soil and natural soil showed 26.6% and 33.4% decrease in the growth of RS (*P* = 0.0001) inoculated on the culture medium compared to control, respectively (Fig. 1D). The VOCs produced in the sterilized soil in control treatment did not show any growth inhibition of RS; however, the VOCs produced in natural soil without the inoculation of SQR-9 (control) showed 7.5% growth inhibition of RS compared to the sterilize soil control.

### Effect of VOCs of SQR-9 on the motility traits, hydrolytic enzymes and antioxidant enzymes of RS

**Figure 2:**
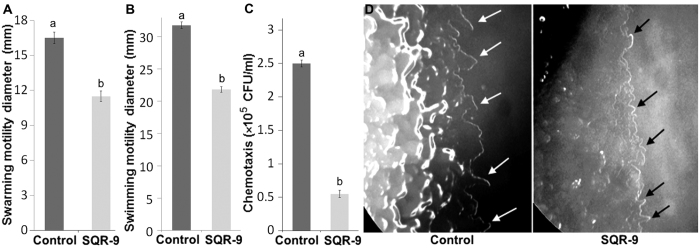
Effect of volatile organic compounds produced by *Bacillus amyloliquefaciens* SQR-9 on the motility traits of *Ralstonia solanacearum.* **(A)** Swarming motility; **(B)** Swimming motility; **(C)** Chemotaxis; **(D)** Twitching motility. White arrows indicate the wider peripheral colony fringe of *Ralstonia solanacearum* in control while black arrows indicate the small peripheral colony fringe of *Ralstonia solanacearum* after exposure to the VOCs of SQR-9. Error bars indicate standard deviations of three replicates and different letters describe significant differences as determined by student’s t-test at *P* = 0.05 within the same data group.

The results of the effect of VOCs on the motility traits of pathogen RS showed that all tested motility traits swarming, swimming, chemotaxis and twitching motility were significantly reduced after exposure to the VOCs of SQR-9 and this reduction was 30%, 31% and 78% after three days for swarming (*P* = 0.0005), swimming (*P* = 0.0001), and chemotaxis motility (*P* < 0.00001), respectively (Fig. 2A–C). In the chemotaxis assay, the RS strain showed motility only towards the hole containing root exudates (10–12 mm in control) while motility in other directions was not found. In the negative control, where sterilized water was used in place of root exudates, the RS strain showed motility in all directions, but only up to 1–2 mm, which clearly differentiated the chemotaxis motility from the general motility (data not shown).

**Figure 3:**
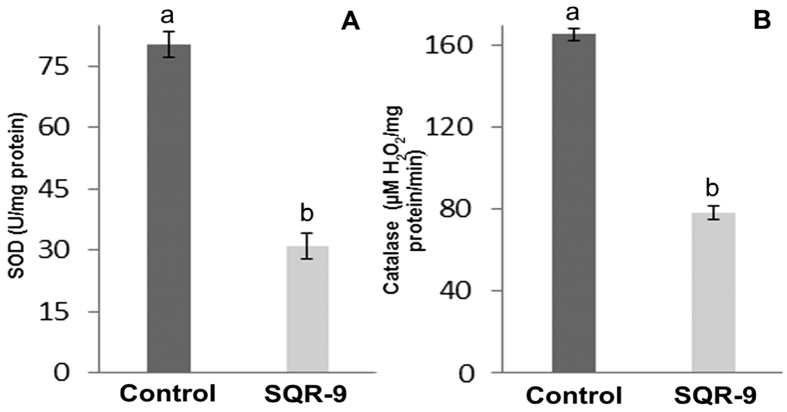
Effect of volatile organic compounds produced by *Bacillus amyloliquefaciens* SQR-9 on the production of antioxidant enzymes: superoxide dismutase **(A)** and catalase **(B)** by tomato wilt pathogen *Ralstonia solanacearum*. Error bars indicate standard deviations of three replicates and different letters describe significant differences as determined by student’s t-test at *P* = 0.05 within the same data group.

The twitching motility results showed that the peripheral colony fringe of RS in control was significantly wider than the VOCs exposed RS colony which showed the reduction in twitching motility of the RS after exposure to the VOCs of SQR-9 (Fig. 2D). In this study, two hydrolytic enzymes were selected to evaluate their production by RS after exposure to VOCs of SQR-9. The results showed that endo-glucanase activity was not changed after exposure to the VOCs; however, a slight but significant decrease of 6% (*P* = 0.0001) in polygalacturonase activity compared to control was observed (data not shown). Similarly, the VOCs of SQR-9 significantly reduced the production of antioxidant enzymes by RS. The SOD activity was decreased by 62% (*P* = 0.0001), while the catalase activity was decreased by 72% (*P* = 0.0009) after three days compared to control (Fig. 3A,B).

### Effect of VOCs of SQR-9 on root colonization, EPS production and biofilm formation by RS

**Figure 4:**
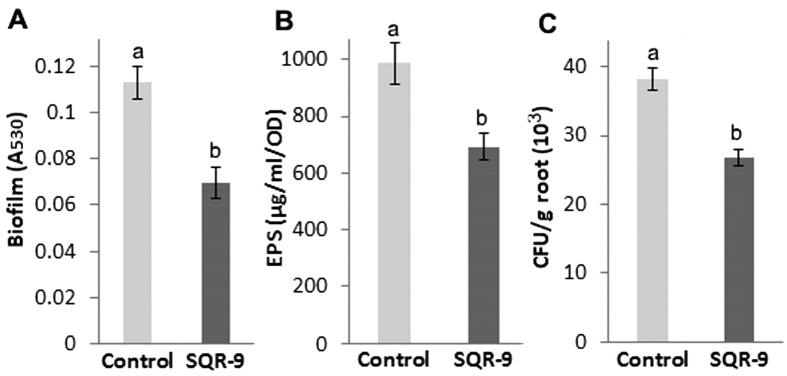
Effect of volatile organic compounds produced by *Bacillus amyloliquefaciens* SQR-9 on biofilm formation **(A)**, exopolysaccharides production **(B)** and tomato root colonization **(C)** by tomato wilt pathogen *Ralstonia solanacearum*. Error bars indicate standard deviations of three replicates and different letters describe significant differences as determined by student’s t-test at *P* = 0.05 within the same data group.

The VOCs produced by SQR-9 showed a significant negative effect on the production of EPS, formation of biofilm and root colonization of tomato roots by pathogen RS. The results showed a 47% decrease (*P* = 0.0008) in the biofilm formation by RS (Fig. 4A), while the decrease in the EPS production (*P* = 0.0098) was 31% after exposure to the VOCs of SQR-9 compared to control (Fig. 4B). The root colonization assay showed that 3.91 × 10^4 ^CFU/g fresh root RS cells colonized tomato roots in control while the root colonization was decreased to 2.87 × 10^4 ^CFU/g fresh root RS cells (*P* = 0.0006) after exposure to the VOCs of SQR-9 (Fig. 4C).

### VOCs produced by SQR-9 and their antibacterial activity

**Figure 5:**
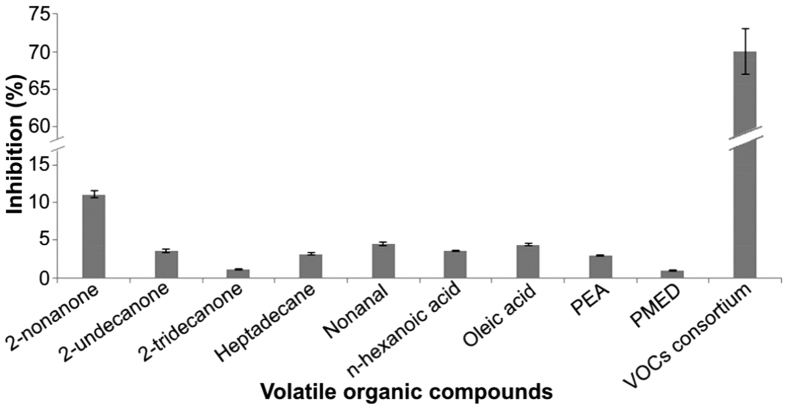
Antibacterial activity of pure volatile organic compounds (VOCs) alone and in consortium against *Ralstonia solanacearum*. Antibacterial activity of all VOCs was assessed in the amounts corresponding to those produced by *Bacillus amyloliquefaciens* SQR-9 and only the VOCs that showed antibacterial activity are presented here. Error bars indicate standard deviations of three replicates. PEM = Phenylethyl alcohol; PMED = Phenol, 4,4′-(1-methylethylidene) bis-.

The strain SQR-9 produced 22 VOCs, including eight ketones, four alkanes, three aldehydes, four acids, and one each alcohol, furan and phenolic compound (Supplementary file Fig. S1). Among those, heptadecane was produced in the maximum amount under the experimental conditions followed by 2-undecanone, undecanal, 2-tridecanone and so on (Table 1). All VOCs were evaluated in their corresponding amounts for the antibacterial activity against RS and only nine VOCs showed antibacterial activity against RS. Among different ketones, only 2-nonanone, 2-undecanone and 2-tridecanone showed 11.1%, 3.6% and 1.1% growth inhibition of RS, respectively. Among the three alkanes, only heptadecane showed a 3.2% decrease in the growth of RS. The aldehydes nonanal and undecanal at their corresponding concentrations showed 4.6% and 3.5% antibacterial activity, respectively, but hexadecanal was not able to inhibit the growth of RS. Among different acids produced by strain SQR-9, n-hexanoic acid and oleic acid showed 3.6% and 4.4% antibacterial activity against RS, respectively. Phenylethyl alcohol and phenol, 4,4′-(1-methylethylidene) bis- showed 3% and 1% antibacterial activity against RS, respectively, while we were not able to purchase furan 2-ethyl-5-methyl so its antibacterial activity was not detected. When the consortium of all VOCs in their corresponding amounts was used, then 70% growth inhibition of RS was determined after three days (Fig. 5).

### Proteomics analysis

The 2D proteomics analysis showed that the expression of 86 proteins was significantly changed after exposure to the VOCs of SQR-9. Among those, 32 proteins were down regulated while the expression of 54 proteins was increased (Supplementary file Fig. S2). A total of 25 proteins that showed more than 2 times changed expression were excised and sequenced. Among those, the expression of 12 proteins was decreased after exposure to the VOCs of SQR-9. These proteins were mainly related to the antioxidant activity, inclusion body proteins, carbohydrate and amino acid metabolism and protein folding and translation. One protein related to the virulence of RS pathogen transcriptional regulator (*PhcA*) was also down regulated. A total of 13 proteins that showed increased expression were mainly related to the ABC transporter system, protein translation and folding, metabolism, detoxification of aldehydes and ketones, biomolecules methylation, and energy transfer (Table 2). These results revealed a significant impact of VOCs produced by SQR-9 on the cellular functions of RS.

## Discussion

In this first study, we reported that the VOCs produced by a biocontrol strain SQR-9 significantly reduced the growth of tomato wilt pathogen RS on agar medium and in soil, and this antibacterial effect was increased with the increasing concentration of strain SQR-9. The control treatments (*E. coli* DH5α and activated charcoal) showed the same results as control, in addition, strain SQR-9 was not able to produce ammonia and cyanide. These results revealed that the growth inhibition of RS was not because of oxygen depletion or ammonia or cyanide production, but was only because of the VOCs of strain SQR-9. The RS cells were able to resume their normal growth after removing strain SQR-9, which revealed the bacteriostatic action of VOCs of SQR-9 on RS. The effect of VOCs produced by biocontrol strains on pathogen RS has not been reported before; however, the VOCs produced by a fungal pathogen *Aspergillus flavus* showed four-fold reduction in the growth of RS^26^. The VOCs of biocontrol strains have shown antibacterial activity against other pathogens. Like the VOCs produced by the *Pseudomonas fluorescens* B-4117 and *Serratia plymuthica* IC1270 inhibited the growth of *Agrobacterium tumefaciens* and *Agrobacterium vitis*^15^. Similarly, the VOCs of *Pseudomonas chlororaphis* 449 and *Serratia proteamaculans* 94 exhibited bacteriostatic effect on phytopathogenic *Agrobacterium tumefaciens* C58^27^.

The VOCs produced by strain SQR-9 on agar medium decreased the population of RS in infected soil; however, this growth inhibition appeared to be lower than the growth inhibition of RS on agar medium. The RS cells might be covered by soil particles which hindered the contact of VOCs with RS cells that caused less growth inhibition of RS compared to that on agar medium. However, this effect might not be significant when both strains SQR-9 and RS would be present close in soil at the micro-environment. The strain SQR-9 was also able to produce antibacterial VOCs when inoculated in sterilized soil and natural soil; however, the VOCs produced in natural soil inoculated with SQR-9 or not (control), both showed more growth inhibition of RS compared to that in sterilized soil that might be the contribution of soil indigenous microbes as many microbial strains isolated from soil have shown the production of antimicrobial VOCs^28^,^29^. Similar results in natural and sterilized soil were reported by Raza *et al.*^21^. On the other hand, the VOCs produced by SQR-9 in sterilized soil appeared to show lower antibacterial activity compared to the agar medium. It might be because the synthetic agar medium has different or easily available nutrients compared to soil as Fiddaman and Rossall^30^ reported that the production of VOCs is significantly influenced by the media contents.

The flagella-mediated motility and chemotaxis attraction toward root exudates are essential for pathogenic RS strains to efficiently colonize and enter into plant roots to cause disease^31^. In addition, the motility in RS is coregulated with several known virulence factors in a regulatory cascade^32^. The VOCs of SQR-9 significantly reduced the motility traits of RS. There is no information available about the effect of VOCs of biocontrol strains on the motility traits of plant pathogens; however, the inhibitory role of *B. subtilis* VOCs on the motility traits of *E. coli* has been reported^33^. In another report, Létoffé *et al.*^34^ suggested that the inhibition of *E. coli* motility upon exposure to VOCs was partly due to the growth inhibition. They also reported that some of the VOCs of *E. coli* decreased while some increased the motility of *Pseudomonas aeruginosa.* The inhibition of motility traits of RS by VOCs of SQR-9 showed the significance of VOCs that might keep pathogen away from the rhizosphere of tomato not only by reducing its growth rate but also by restricting its movement to invade plant roots.

The RS strains produce a consortium of extracellular hydrolytic enzymes that facilitate bacterial invasion and spread by digesting cortical cell wall and are known to be required for virulence by RS^35^. The results of this study showed that the VOCs of SQR-9 did not inhibit the production of endo-glucanase, but slightly decreased the polygalacturonase activity. Much information is not available about the effect of VOCs on hydrolytic enzymes; however, the effect of VOCs on other microbial enzymes has been reported like Fialho *et al.*^36^ reported that *Saccharomyces cerevisiae* CR-1produced VOCs that significantly inhibited the production of morphogenesis-related enzymes in *Guignardia citricarpa*. The VOCs produced by SQR-9 also showed a significant negative effect on the production of EPS, formation of biofilm and tomato root colonization by pathogen RS. The decrease in root colonization by RS might be because of decrease in the ability of biofilm formation, which is also related to the EPS production. The EPS production by RS is a well characterized bacterial wilt virulence factor and EPS-deficient strains colonize plants poorly and cause little or no disease^37^. Another reason might be that the RS cells modified themselves for survival under stress conditions imposed by the VOCs of SQR-9 rather than preparing for root colonization. Similar to our results were reported earlier, when the VOCs of *Aspergillus Flavus* showed five-fold reduction in the EPS production by RS^26^. Létoffé *et al.*^34^ reported the concentration dependent inhibition of biofilm formation by *Pseudomonas aeruginosa*, *Staphylococcus aureus* and *Bacillus subtilis* after exposure to the VOCs of *E. coli*.

In this study, the exposure of VOCs of SQR-9 decreased the SOD and catalase activity of RS more than two times compared to control. These results were not in agreement with that of Fialho *et al.*^38^ who reported that the SOD and catalase activity of *Guignardia citricarpa* was increased after exposure to the VOCs of *Saccharomyces cerevisia*. The RS shows antioxidant activity under normal conditions as the reactive oxygen species (ROS) are generated during respiratory metabolism and RS may constitute an adaptive response to the enhanced generation of ROS in entering into the stationary phase as observed in *Saccharomyces cerevisia*^39^. In addition, antioxidant enzymes are important for phytopathogens not only to neutralize their own ROS, but also to counteract the oxidative burst generated by their host plants during the initial phase of infection. Therefore, antioxidant enzymes are considered an important virulence factor for phytopathogens^40^. The decrease in antioxidant enzyme production might be because of a reduction in the metabolic or respiratory activity of RS cells after exposure to the VOCs of SQR-9 or VOCs themselves acted as antioxidant agents, as the sulfur compounds, terpenes, phenols, esters, aldehydes, alcohols and glycosides have shown antioxidant functions^41^.

The VOCs of microbes are most likely produced by the modification of products of the fatty acid biosynthetic pathway and shikimate pathway^42^. The VOCs of SQR-9 showed 1–11% growth inhibition of RS in their corresponding produced amounts. Although, the VOCs produced by SQR-9 have been reported to be produced by other microbial strains and have shown antimicrobial activity^21^,^28^. However, in this study, the concentration of individual VOC produced by SQR-9 might be low for higher antibacterial activity against the RS. The concentration of VOCs of SQR-9 might be different in soil and with organic fertilizer as carrier material^21^. In addition, toxins produced by pathogens also affect the production of VOCs in soil^28^. When the consortium of VOCs of SQR-9 in their corresponding amounts was used, then 70% growth inhibition of RS was determined, which was 2.5 times higher than the growth inhibition of RS in the plate assay. The reason might be that the production of VOCs was increased gradually with the growth of SQR-9 which gave time RS to grow up to some extent, but the use of consortium of all VOCs at the initial growth stage of RS did not give it enough time to grow well, which increased the growth inhibition rate of RS.

The proteomics analysis showed that the proteins involved in antioxidant activity were downregulated like thiol peroxidase, catalase and polyphenol oxidase. The pathogen RS produces antioxidant enzymes not only to neutralize its own ROS but also to counter the oxidative burst of the host plants. The decrease in antioxidant enzyme production might be because of a reduction in the metabolic or respiratory activity of RS cells after exposure to the VOCs of SQR-9 or VOCs itself acted as antioxidant agents which decreased the production of antioxidant enzymes. The antioxidant potential of VOCs like aldehydes, ketones which were also produced by strain SQR-9, has been reported before^41^. The proteins chain A, lectin (Rs-Iil) with α-methylmannoside and tryptophan 2-monooxygenase oxidoreductase which are involved in carbohydrates binding and metabolism and tryptophan metabolism, respectively, were downregulated, which indicates the toxic effect of VOCs on the cells of RS. There was one inclusion body protein and three hypothetical proteins, which were downregulated. The inclusion body proteins are nuclear or cytoplasmic aggregates of substances and among hypothetical proteins, one protein also showed homology to the inclusion body protein, while one showed homology to the type IV secretion system protein, which is involved in the secretion of macromolecules and virulence factor proteins directly into the host cell and vesicular transport^43^. One hypothetical protein did not show any homology and was conserved in RS. The molecular chaperone *GroEL* protein, which in involved in protein folding, and protein chain elongation factor EF-Tu that helps the aminoacyl-tRNA to move on a free site on the ribosome were downregulated. Our results were not in agreement with Hennequin *et al.*^44^ who reported that *GroEL* protein expression was induced under adverse environmental conditions. The downregulation of one important virulence related protein *PhcA* was also detected, which is an important *LysR*-type transcriptional regulator and induces the EPS, β-1, 4-endoglucanase and pectin methylesterase production at high cell densities^45^. In this study, the decrease in EPS and polygalacturonase production by RS after exposure to the VOCs of SQR-9 also supported these results.

Among the proteins which were upregulated by the VOCs of SQR-9, three proteins were belonged to the ABC transporter system, one was specific to phosphate transport and two belonged to the binding proteins. The ABC transport system is an important part of the bacterial cell and is involved in the translocation of various substrates across membrane and non-transport related processes like translation of RNA, DNA repair and antibiotic resistance^46^. In addition, ABC transporter proteins are also reported to play a role in the EPS synthesis^47^ and are required for cell integrity and cell survival^48^. The VOCs have been reported to cause structural changes on the cell envelop or even damaged the cell wall and ABC transporter proteins overexpression might be because of their role in the osmo-adaptation and maintenance of cell integrity and survival in response to undesirable change occurring to cell. This conclusion was also supported by the increased expression of hypothetical membrane spanning protein, which is supposed to be involved in the cell integrity. The increased expression of ABC transporter proteins under oxidative and osmotic stress in *Streptococcus mutans* has been reported by Nagayama *et al.*^49^. Three proteins related to the protein translation and folding were over expressed which might be because of cell requirements for the expression of some stress related proteins or epigenetics modifications under stress conditions imposed by the VOCs. Electron transfer flavoprotein subunit beta protein, which is involved in the process of electron acceptance for dehydrogenases, transfer of electrons to terminal respiratory systems and fatty acid oxidation was upregulated. It might be partially related to the over expression of aldehyde dehydrogenase, amino acid biosynthesis proteins like succinyl-CoA-3-ketoacid-CoA transferase and other stress related proteins in this study that require electrons for functioning. The electron transfer flavoprotein subunit beta proteins are also involved in the fatty acid oxidation which might be the response of RS cells by producing antibacterial VOCs in response to the VOCs of SQR-9. In addition, aldehyde dehydrogenase and succinyl-CoA-3-ketoacid-CoA transferase proteins were upregulated, which detoxify exogenously and endogenously generated aldehydes and ketones, respectively, as the aldehyde and ketone group VOCs were produced by strain SQR-9. Another reason might be that when the uptake of outer energy sources was compromised under stress conditions, succinyl-CoA-3-ketoacid-CoA transferase helped RS cells to utilize internal energy sources as reported by White & Jencks^50^.

Two proteins related to the isomerization, 1-(5-phosphoribosyl)-5-[(5-phosphoribosylamino) methylideneamino] imidazole-4-carboxamide isomerase which is involved in L-histidine biosynthesis; and putative isomerase rotamase signal peptide protein which accelerates the folding of proteins by catalyzing the cis-trans isomerization were upregulated. The isomerases are involved in intramolecular rearrangements and catalyze conformational changes^51^. Their increased expression might be the results of toxic effects of VOCs on RS and cells endured rearrangements and conformational changes. Another important protein S-adenosylmethionine-dependent methyltransferase was upregulated, which plays a critical role in the cell development, functions, virulence and survival by the methylation of biomolecules and secondary metabolites. Its upregulation shows the methylation of biomolecules under stress conditions as the protecting role of methytranferases against oxidative stress in *Podospora anserina* has been reporter earlier^52^.

This is the first report of the effect of VOCs produced by a biocontrol strain SQR-9 on the growth and virulence traits of tomato wilt pathogen RS. The results of this study clearly revealed the importance of VOCs in the control of plant pathogens. In contrast to the antibiotics, which can only prevent pathogens from infecting plants if biocontrol agents colonize directly onto the plant roots, the VOCs can spread over a long distance and bacteriostatic microenvironment exists around the antagonist communities^53^. The VOCs of SQR-9 would play an important role to keep pathogen away from the rhizosphere of tomato not only by reducing its growth rate, but also by restricting its movement to invade plant roots and inhibiting other important virulence traits; however, the concentration of biocontrol agent will be critical for its effectiveness. Information on the action mechanisms is important to better understand the microbial interactions mediated by VOCs in nature and to develop safer strategies to control plant disease.

## Additional Information

**How to cite this article**: Raza, W. *et al.* Response of tomato wilt pathogen Ralstonia solanacearum to the volatile organic compounds produced by a biocontrol strain Bacillus amyloliquefaciens SQR-9. *Sci. Rep.* **6**, 24856; doi: 10.1038/srep24856 (2016).

## Supplementary Material

Supplementary Information

## Figures and Tables

**Table 1:** The relative peak area and amount of volatile organic compounds produced by *Bacillus amyloliquefaciens* SQR-9.

RT	Volatile organic compounds	Relative peak area (%)	Production (μg)
12.913	2-nonanone	4.41 ± 0.19	4.15 ± 0.59
14.158	2-decanone	0.44 ± 0.04	0.25 ± 0.08
17.20	2-undecanone	11.1 ± 0.29	9.14 ± 0.55
20.267	2-dodecanone	2.83 ± 0.08	1.2 ± 0.38
21.763	2-tridecanone	7.83 ± 0.59	5.25 ± 1.20
16.432	2-tetradecanone	4.23 ± 0.19	3.2 ± 0.61
18.056	2-pentadecanone	1.35 ± 0.05	1.0 ± 0.28
18.529	2-nonadecanone	3.74 ± 0.59	1.8 ± 0.37
24.444	Heptadecane	13.1 ± 0.18	12.6 ± 1.5
15.287	Pentadecane	5.41 ± 0.25	4.7 ± 0.63
12.156	Dodecane	1.23 ± 0.05	0.5 ± 0.15
12.505	Tridecane	0.98 ± 0.02	1.25 ± 0.10
10.995	Nonanal	0.29 ± 0.01	0.2 ± 0.01
14.411	Undecanal	9.16 ± 0.24	7.75 ± 0.38
14.65	Hexadecanal	1.55 ± 0.09	1.0 ± 0.21
20.951	n-hexanoic acid	4.99 ± 0.16	1.75 ± 0.38
26.151	Hexadecanoic acid	5.54 ± 0.19	2.5 ± 0.29
28.221	Trans-13-octadecanoic acid	0.74 ± 0.05	0.45 ± 0.02
28.453	Oleic acid	1.00 ± 0.04	0.5 ± 0.07
13.462	Phenylethyl alcohol	0.98 ± 0.01	0.95 ± 0.12
8.076	Furan 2-ethyl-5-methyl	0.72 ± 0.01	–
28.584	Phenol, 4,4′-(1-methylethylidene) bis-	1.85 ± 0.01	1.1 ± 0.24

The values after ± indicate the standard deviations. The peak area was calculated in percentage over the total area of all peaks. The pure standard compounds were purchased from Sigma, Tokyo Chemical Industry Co., Ltd. (TCI, Japan) and Aladdin Reagent Database, Inc. (Shanghai, China). RT = retention time.

**Table 2:** The identified proteins of *Ralstonia solanacearum* which showed more than two times change in the expression level after exposure to the volatile organic compounds produced by *Bacillus amyloliquefaciens* SQR-9.

Spot	Protein name	Accession no.	PS	MP	SC (%)	MW (KDa)/pI	Reg.
1	Chain A, Lectin (Rs-Iil) with Alpha-methylmannoside	48425832	410	48	42	11.6/4.36	Down
2	Hypothetical protein	515094274	136	29	15	19.9/4.57	Down
3	Thiol peroxidase	525967097	513	87	52	17.4/5.37	Down
5	Inclusion body protein	515093205	171	62	34	18.9/6.56	Down
6	Hypothetical protein	515093206	419	101	56	19.1/5.72	Down
7	Hypothetical protein	518547205	557	77	28	30.2/6.10	Down
8	Molecular chaperone *GroEL*	523414217	550	101	18	57.5/5.09	Down
10	Protein chain elongation factor EF-Tu	299077198	482	86	21	43.2/5.43	Down
11	Polyphenol oxidase B precursor MG12	222875587	261	46	9	52.6/8.88	Down
12	Catalase	469777473	908	145	29	55.4/7.25	Down
13	Tryptophan 2-monooxygenase oxidoreductase	499312265	762	126	17	76.1/6.86	Down
28	Transcriptional regulator (*PhcA*)	309881	95	29	8	38.5/6.02	Down
14	Translation elongation factor Tu	147752135	128	40	10	43.4/5.43	Up
15	Succinyl-CoA–3-ketoacid-CoA transferase	499310740	155	29	13	22.5/4.86	Up
16	1-(5-phosphoribosyl)-5-[(5-phosphoribosylamino) methylideneamino] imidazole-4-carboxamide isomerase	499312077	195	38	15	26.5/4.80	Up
17	Elongation factor P (EF-P)	299079026	135	34	18	21.2/4.98	Up
19	Hypothetical membrane spanning protein	83725915	88	23	4	52.4/9.06	Up
20	Translation elongation factor Tu domain II	221166965	68	31	8	20.5/5.16	Up
21	Putative isomerase rotamase signal peptide protein	17428761	288	65	25	28.0/6.25	Up
22	S-adenosylmethionine-dependent methyltransferase	655479450	539	89	32	31.2/5.65	Up
23	Electron transfer flavoprotein subunit beta (FAD-binding domain)	499310101	208	54	16	26.7/5.10	Up
24	ABC transporter permease (ligand binding cite)	499310903	271	41	12	34.2/9.34	Up
25	Phosphate-binding protein PstS precursor (PBP), ABC transporter	299078282	836	102	29	36.3/9.28	Up
26	ABC transporter permease (Periplasmic binding protein)	499311995	343	78	20	40.6/9.33	Up
27	Aldehyde dehydrogenase	499312243	231	63	12	55.4/5.95	Up

PS = protein score; MP = matched peptides; SC = sequence coverage; pl = isoelectric point; Reg. = regulation.
