# Supplementary material for: Response of tomato wilt pathogen Ralstonia solanacearum to the volatile organic compounds produced by a biocontrol strain Bacillus amyloliquefaciens SQR-9
Source: Sci Rep. 2016 Apr 22;6:24856. doi: 10.1038/srep24856 (PMC4840334; doi:10.1038/srep24856)
Supplement: Supplementary Information [file srep24856-s1.pdf]

Supplementary material

Response of tomato wilt pathogen *Ralstonia solanacearum* to the volatile organic compounds produced by a biocontrol strain *Bacillus amyloliquefaciens* SQR-9

Waseem Raza, Ning Ling, Liudong Yang, Qiwei Huang, Qirong Shen*

Jiangsu Collaborative Innovation Center for Solid Organic Waste Utilization, College of Resources and Environmental Sciences, Nanjing Agricultural University, Wei Gang Road, No. 1, 210095, Nanjing, Jiangsu Province, P. R. China

***Corresponding author** Qirong Shen

Complete postal address Jiangsu Collaborative Innovation Center for Solid Organic Waste Utilization, College of Resources and Environmental Sciences, Nanjing Agricultural University, Wei Gang Road, No. 1, 210095, Nanjing, Jiangsu Province, P. R. China.

Phone number 0086-13901586468

E-mail address shenqirong@njau.edu.cn

Fax number 0086-2584432420

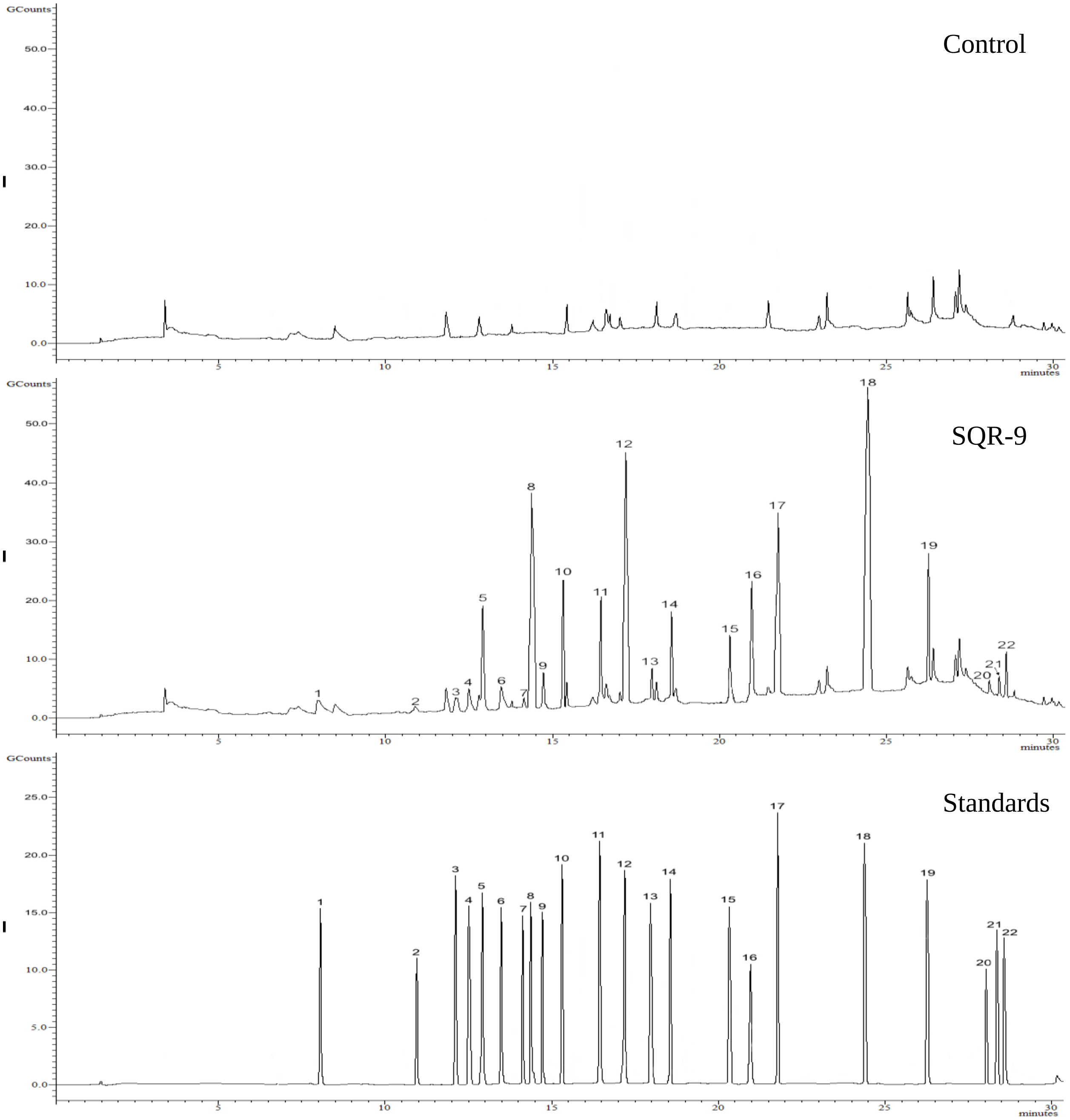

Figure S1: GC chromatograph of volatile organic compounds in control, produced by strain *Bacillus amyloliquefaciens* SQR-9 and standard volatile organic compounds.

1= Furan 2-ethyl-5-methyl; 2 = Nonanal; 3 = Dodecane; 4 = Tridecane; 5 = 2-nonanone; 6 = Phenyl ethyl alcohol; 7 = 2-decanone; 8 = Undecanal; 9 = Hexadecanal; 10 = Pentadecane; 11 = 2-tetradecanone; 12 = 2-undecanone; 13 = 2-pentadecanone; 14 =2-nonadecanone; 15 = 2-dodecanone; 16 = n-hexanoic acid; 17 = 2-tridecanone; 18 = Heptadecane; 19 = Hexadecanoic acid; 20 = Trans-13-octadecanoic acid; 21 = Oleic acid; 22 = Phenol 4, 4-(1-methylethylidene) bis

The pure standard compounds were purchased from Sigma, Tokyo Chemical Industry Co., Ltd. (TCI, Japan) and Aladdin Reagent Database, Inc. (Shanghai, China).

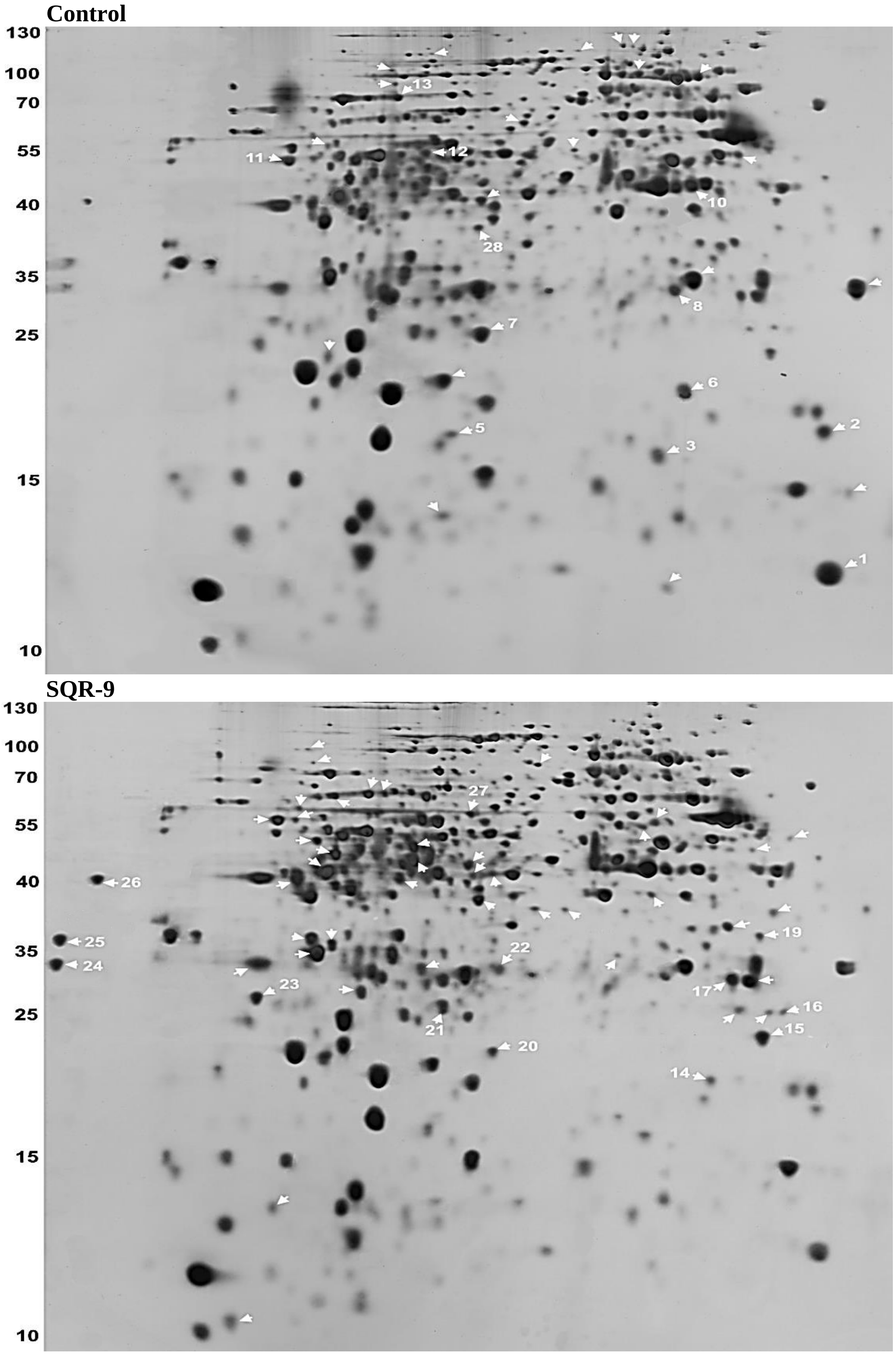

Figure S2: 2D analysis of cellular proteins of *Ralstonia solanacearum* with and without exposure to the volatile compounds produced by *Bacillus amyloliquefaciens* SQR-9. The proteins marked with white arrows indicate the change in expression levels while numbered proteins indicated increased expression levels by two or more than two times in corresponding treatment. The numbered proteins were sequenced for identification. Numbers of the left side indicate the protein markers (KDa).
